# Availability, consistency and evidence-base of policies and guidelines on the use of mask and respirator to protect hospital health care workers: a global analysis

**DOI:** 10.1186/1756-0500-6-216

**Published:** 2013-05-31

**Authors:** Abrar Ahmad Chughtai, Holly Seale, Chandini Raina MacIntyre

**Affiliations:** 1 School of Public Health and Community Medicine, UNSW Medicine, University of New South Wales, Sydney2052, Australia; 2 National Centre for Immunization Research and Surveillance of Vaccine Preventable Diseases (NCIRS), The Children’s Hospital, Westmead, Australia

**Keywords:** Infectious diseases, Seasonal influenza, Pandemic influenza, Sever Acute Respiratory Syndrome (SARS), Tuberculosis (TB), Masks, Respirators

## Abstract

**Background:**

Currently there is an ongoing debate and limited evidence on the use of masks and respirators for the prevention of respiratory infections in health care workers (HCWs). This study aimed to examine available policies and guidelines around the use of masks and respirators in HCWs and to describe areas of consistency between guidelines, as well as gaps in the recommendations, with reference to the WHO and the CDC guidelines.

**Methods:**

Policies and guidelines related to mask and respirator use for the prevention of influenza, SARS and TB were examined. Guidelines from the World Health Organization (WHO), the Center for Disease Control and Prevention (CDC), three high-income countries and six low/middle-income countries were selected.

**Results:**

Uniform recommendations are made by the WHO and the CDC in regards to protecting HCWs against seasonal influenza (a mask for low risk situations and a respirator for high risk situations) and TB (use of a respirator). However, for pandemic influenza and SARS, the WHO recommends mask use in low risk and respirators in high risk situations, whereas, the CDC recommends respirators in both low and high risk situations. Amongst the nine countries reviewed, there are variations in the recommendations for all three diseases. While, some countries align with the WHO recommendations, others align with those made by the CDC. The choice of respirator and the level of filtering ability vary amongst the guidelines and the different diseases. Lastly, none of the policies discuss reuse, extended use or the use of cloth masks.

**Conclusion:**

Currently, there are significant variations in the policies and recommendations around mask and respirator use for protection against influenza, SARS and TB. These differences may reflect the scarcity of level-one evidence available to inform policy development. The lack of any guidelines on the use of cloth masks, despite widespread use in many low and middle-income countries, remains a policy gap. Health organizations and countries should jointly evaluate the available evidence, prioritize research to inform evidence gaps, and develop consistent policy on masks and respirator use in the health care setting.

## Introduction

To maintain the functionality and capacity of the healthcare workforce during outbreaks or pandemics of emerging infections, such as influenza, health care workers (HCWs) need to be protected. Medical masks (“masks”) and respirators are commonly used to protect HCWs from respiratory infections. In the healthcare setting, masks are used to prevent HCWs acquiring respiratory infections, from splashes of blood and body fluids and to reduce transfer of potentially infectious body fluids in the sterile area. Alternatively, they may be used by the HCW and coughing patient to prevent the spread of infection in the ward, referred to as “source control” [1-4]. Masks were not designed to provide respiratory protection [5], as they have consistently lower filtration efficiency than respirators [6-9]. A respirator is a fitted device that protects the wearer against inhalation of small and large airborne particles, that is, it protects the wearer from others who are or might be infected [2].

High-income countries have established infection control programs which can be implemented with good resourcing. The guidelines and advice underlying these control programs have been produced by high-income countries for their own social, economic, and health environments. Low and middle income countries may not have the ability or finances to adopt generic infection control or pandemic guidelines, equivalent to those originating from high income countries. The practices occurring in low/middle income countries may be driven by a number of factors other than available scientific evidence – such as available resources, Occupational Health and Safety (OHS) legislation, culture, logistics and cost considerations.

Whilst much has been written about available policies issued by the World Health Organization (WHO), and the United States Centers for Disease Control and Prevention (CDC), little is known about the consistency in policies from low and middle income countries, and country-specific issues which can drive different needs. In light of ongoing threats from influenza (H1N1, H5N1 and H7N9) and other emerging infections, it is essential to examine the policies and guidelines of various organizations and countries to examine whether they are evidence based, and whether there are any issues with the recommendations. This study aimed to examine available policies and guidelines around the use of masks and respirator for HCWs, for the prevention of influenza, SARS and TB; and to describe areas of consistency and inconsistency between guidelines, as well as gaps, with reference to the WHO and the CDC guidelines.

## Methods

The guidelines of two large public health organizations, three high-income countries and six low/middle income countries were purposely selected for inclusion in this study. We included guidelines from two major health organizations which are commonly used internationally as a reference, namely the World Health organization (WHO) and the US Centers for Disease Control (CDC). Guidelines from three high income countries (Australia, Canada and UK) and six middle/low income countries (Bangladesh, China, India, Indonesia, Pakistan and Vietnam) were also selected. The main reasons for purposively selecting these guidelines was that the six low/middle income countries account for 47% of the world’s population and represent areas where emerging infectious diseases are likely to arise from. Most of these guidelines were publically available or were accessed through known key contacts, and were available in a language which could be readily translated in-house.

We selected guidelines related to influenza, SARS and TB for this review. Given that influenza has the potential to cause both seasonal infections and pandemics; it was chosen as the primary infection of interest. TB was selected as an example of a chronic but highly infectious disease. In contrast to influenza, TB has a long incubation and infection period. Lastly, SARS was selected as an example of emerging infectious disease, which required a rapid response.

### Search strategy

Information relating to mask and respirator use was extrapolated from the following sources: a) general infection control guidelines; b) disease specific infection control guidelines (influenza, SARS and TB); c) personal protective equipment guidelines; d) mask/respirator use guidelines and e) position statements. Documents published in the last twelve years in any language were screened with key words for applicability. In the event that two versions of the guideline were found, the most recent version was included. Four strategies were utilized to locate relevant documents. Firstly, websites including the WHO (plus regional offices), CDC, selected countries health departments and other relevant websites were screened. Secondly, a key word search was conducted using Google, with 10 results per page set and the first two pages of hits reviewed. The policies and guidelines were also searched in the native languages of the selected countries through advance search settings in Google. The search results were narrowed down by selecting region (e.g. India), site or domain (e.g. gov) and file type (e.g. pdf). Google translator was used to screen the documents in the native languages and then the selected documents were translated by native language speaking colleagues. Policies and guideline documents were also searched for using Medline, Embase, National Guidelines Clearinghouse, and Google Scholar through key words. Lastly, key personal contacts in the selected countries were contacted in regards to the availability of guidelines in the country. Most of the contacts are employed in Government organizations or health institutions.

### Collection and analysis

The predefined criteria were used to screen the guidelines for their eligibility. Title and summaries were firstly assessed by AAC and then validated by HS and CRM. The following information was extracted from each of the selected guidelines; country/organization, department, publication year, language, title and recommendation on mask/respirator use. The terminology used in different countries and guidelines varied, so a classification system was devised (Table 1).

**Table 1:** Terminology used in guidelines reviewed

**Terminology**	**Classification for this study**
Mask; surgical mask; medical mask; procedure mask.	Mask
N95; N99; N100; FFP1; FFP2; FFP3; P1; P2; P3; particulate respirator.	Respirator
**Low risk situations described in influenza and SARS guidelines:** Close contact within one meter of the patient, close contact within 2 meters of the patient; entering infectious patient’s room; clinical care; all patients contact; when infected patient used masks; routine care; in screening area; during patients transport; before and after patients contact and risk of splashes into face.	Low risk situations
**Low risk situations described in TB guidelines:** Low risk facilities including sputum microscopy centers; district and sub district level hospitals.	
**High risk situations described in influenza and SARS guidelines:** Aerosol generating procedures (AGPs); procedures involving the respiratory tract; laboratory specimen collection from respiratory tract; if patients cough forcefully; if patients do not comply with respiratory hygiene; when patients may not be able to wear mask; mortuary and critical care areas.	High risk situations
**High risk situations described in TB guidelines:** Exposure to drugs resistant organism; culture/DST and other high risk procedures in laboratory, high risk areas; specialized treatment centers and emergency surgery of infectious cases.	

## Results

In most of the guidelines reviewed, the rationale for the recommendations around mask and/or respirator use is not discussed and evidence is rarely provided. The WHO, the CDC and most of the countries recommend masks and/or respirator on the basis of the mode of transmission of influenza, SARS and TB. However, various types of masks and respirators are recommended in the guidelines for low and high risk situations. Although most of the guidelines discuss the importance of training and fit testing for respirators use, very few documents provide detail on those procedures. Furthermore, most guidelines do not discuss recommendations on how long masks and respirators should be used for and whether reuse is recommended. Only a few mentioned that a single mask could be used for 4 hours [10], 8 hours [11], or even for an entire shift [12]. Although cloth masks are also commonly used in resource limited settings, the use and reuse of cloth masks is not discussed in any guideline.

A lack of consistency was identified in regards to the nomenclature used in the documents. The WHO frequently uses the term “medical masks” [13], while the CDC uses the term “facemask”. Various terms were also used in the country specific guidelines reviewed. For example, Pakistan uses medical masks, surgical masks is used in the UK, Canada, Australia and India document, procedure masks also in the Canadian document and finally facemasks is the term used in Vietnam. The description of low and high risk situations also varied among the general and disease specific infection control guideline (Table 1).

For seasonal influenza, the WHO [14] and the CDC [15] recommends that masks be used in low risk situations and respirators in high risk situations. The recommendations from the UK [16], Australia [17], India [18] and Pakistan [19] are aligned with those from the WHO and the CDC. However Canada [20] and Vietnam [12] have a different policy, which recommends masks in both low and high risk situations for seasonal influenza. Regarding the choice of respirator, the WHO, the CDC and most of the selected countries recommend an N95 or its equivalent (FFP2 or P2) respirator for seasonal influenza. The UK, however, recommends FPP3 respirators.

Though the WHO and the CDC have the same policy for seasonal influenza, they differ in their recommendations for pandemic influenza. During an influenza pandemic, the WHO recommends mask use in low risk situations and respirators in high risk situations [14], whereas, the CDC recommends respirators in both situations [21]. The guidelines of the UK [22], Canada [23], Australia [4], China [11], India [18] and Pakistan [19] are aligned with those of the WHO (Table 2). For pandemic influenza, the WHO recommends a range of respirators (e.g. P2, P3, FFP2, FFP3, N95, N99 and N100) and the CDC recommend N95 or higher respirators. Canada and most of the low/middle income countries recommend N95 or its equivalent respirators. The UK recommends only FFP3, while Australia recommends P2 or Powered Air Purifying Respirator (PAPR).

**Table 2:** Policies and guidelines on masks and respirators use by health care workers (HCWs)

**Organization/country**	**Seasonal influenza**		**Pandemic influenza**		**SARS**		**TB**	
	**Low risk**	**High risk**	**Low risk**	**High risk**	**Low risk**	**High risk**	**Low risk**	**High risk**
WHO [14,30]	Mask (Medical mask)	Respirators (N95)	Mask (Medical mask)	Respirators (P2/3, FFP2/3, N95/99/100)	Mask (Medical mask)	Respirators (N95)	Respirators (N95, FFP2)	Respirators (N95, FFP2)
CDC, [15,21,24,31]	Mask (Facemasks)	Respirators (N95) or equivalent respirator (e.g., PAPR, elastomeric)	Respirators (N95)	Respirators (N95 or higher respirators)	Respirators (N95)	Respirators (N95 or higher, elastomeric or PAPR)	Respirators (N95)	Respirators (N95 or preferably PAPR)
UK [16,22,25,34]	Mask (Surgical mask)	Respirators (FFP3)	Mask (Surgical mask)	Respirators (FFP3)	Respirators (FFP3), PAPR use is discouraged	Respirators (FFP3), PAPR use is discouraged	Not recommended	Respirators (FFP3)
Canada [13,20,26,32]	Mask (Surgical or procedure mask)	Mask (Surgical or procedure mask)	Mask	Respirators (N95)	Respirators (N95), PAPR use is discouraged	Respirators (N95), PAPR use is discouraged	Respirators (N95)	Respirators (N95)
Australia [4,17,27]	Mask (Surgical mask)	Respirators (P2 or N95)	Mask (Surgical mask)	Respirators (P2 or PAPR)	Respirators (P2 or N95)	Respirators (PAPR)	Respirators (P2, N95)	Respirators (P2, N95)
China [10,11,33]	Guidelines not located	Guidelines not located	Mask (Surgical or medical mask)	Respirators	Mask (Medical mask)	Respirators	Respirators (N95)	Respirators (N95)
India [18,35]	Mask (Surgical mask)	Respirators (N95)	Mask (Surgical mask)	Respirators (N95)	Guidelines not located	Guidelines not located	Not recommended	Respirators (N95 or FFP2)
Indonesia [39]	Guidelines not located	Guidelines not located	Not discussed in pandemic plan	Not discussed in pandemic plan	Guidelines not located	Guidelines not located	Guidelines not located	Guidelines not located
Pakistan [19,28,36]	Mask (Medical mask)	Respirators (N95, FFP2)	Mask (Medical mask)	Respirators (N95, FFP2)	Respirator (N95 or P2)	Respirator (N95 or P2)	Not recommended	Respirators (N95 or FFP2)
Bangladesh [37,40]	Guidelines not located	Guidelines not located	Not discussed in pandemic plan	Not discussed in pandemic plan	Guidelines not located	Guidelines not located	Not recommended	Respirators (N95 or FFP2)
Vietnam [12,29,38,41]	Mask (Facemask)	Mask (Facemask)	Appropriate selection between masks and respirators	Appropriate selection between masks and respirators	Respirator (N95)	Respirator (N95)	Not recommended	Respirators (N95)

The WHO and the CDC have different policies when coming in contact with a patient with SARS. The WHO recommends masks in low risk situations and respirators in high risk situations [14], whereas the CDC recommends that respirators be used in both low and high risk situations [24]. The UK [25], Canada [26], Australia [27], Pakistan [28] and Vietnam [29] also recommend respirators be used by HCWs for protecting themselves from SARS. Only China has the same policy as the WHO [10] (Table 2). The CDC and most of the countries prefer N95 or equivalent respirators in low risk situations in SARS, while the UK recommends a FFP3.

Respirators are recommended by the WHO [30] and the CDC [31] for protection against TB for HCWs in both low and high risk situations. Canada [32], Australia [17] and China [33] have the same policy as previously outlined. In contrast, respirators are recommended only in certain high risk situations in the UK [34], India [35], Pakistan [36] Bangladesh [37] and Vietnam [38] (Table 2). The WHO and most of the selected countries recommend N95 or equivalent respirators for HCWs during low and high risk exposure to TB bacillus. Though the CDC also recommends N95 respirators in low risk situation, elastomeric respirators or PAPR are preferred during the high risk procedures (Table 2).

The seasonal influenza guidelines of China, Indonesia and Bangladesh, SARS guidelines of India, Indonesia and Bangladesh and TB guidelines of Indonesia could not be located; and pandemic guidelines of Indonesia [39] and Bangladesh [40] and Vietnam [41] did not make clear recommendation on masks and respirator use.

Almost all guidelines emphasized the importance of hand hygiene and strongly recommended HCWs to wash their hands before and after patients’ contact to prevent the spread of respiratory infections. The role of other PPEs was also discussed in most of the guidelines. The WHO and the CDC recommended gloves, gown and goggles for seasonal influenza and pandemic influenza in accordance with the standard precaution, i.e. while in contact with infectious material or risk splash on face or body [14,15,21]. However in the case of SARS and other newly emerging infections, both organizations strongly recommended the use of gloves, gown and goggles in all patient contact [14,24].

## Discussion

Considerable variation was observed amongst the policies and guidelines of the selected health organizations and countries in regards to the use of masks and respirators. The WHO and the CDC have a similar policy for seasonal influenza and TB; however, they have different recommendations when dealing with pandemic influenza and SARS. There is also a vast amount of variation between the various country recommendations for the three diseases. We found that influenza related policies of the selected countries were generally in line with the WHO, while SARS related policies were aligned with those from the CDC. The exceptions were the seasonal influenza policies of Canada and Vietnam and the Chinese SARS policy. The previous experience of these three countries with SARS may be a factor influencing the variation in recommendations. TB related policies of high-income countries are in line with the WHO and the CDC, however the policies of the low/middle-income countries are not consistent with either organization.

Various terms were also used in the guidelines reviewed in relation to the products. This indicated that there is no standard terminology or classification for masks. Although the general term “respirator” is constantly used in the guidelines, products with various filtration capacities were recommended for the same diseases. This was especially apparent with regards to the selection of respirators for use during high risk procedures. In some cases, a particular type of respirator recommended by one country was actually discouraged by another country. For example, the CDC and Australia recommend PAPR for high risk situations during SARS, whereas, Canada and UK discourage PAPR use due to the risk of self-contamination [25,26]. Elastomeric respirators or PAPR were only recommended for use by the CDC and the high income countries.

The availability of resources/funding and more stringent OHS regulations in these high-income settings may be factors influencing this trend. Aside from the variation in terminology previously described, some low and high risk situations were classified in a different way. For example, the CDC and Canada recommend respiratory protection within 2 meters of an influenza case, which is different from the WHO policy (1 meter). OSHA also recommends a 2 meter distance [42]. The rationale for 2 meters is not provided in either guideline. Similarly, the Canadian pandemic plan considers it high risk if patients cough forcefully, and/or if patients do not comply with respiratory hygiene [23] and the Australian pandemic plan defines high risk when an infected patient may not able to use masks [4]. However, neither plan provides evidence to support these recommendations.

The WHO and all selected countries have the same policy for pandemic influenza, as for seasonal influenza. The WHO policies are flexible and probably take into account the possibility of resource issues which could occur. In comparison, the CDC policy is different from the WHO and other countries. Due to a lack of pre-existing immunity to pandemic influenza strains, and the potential for the occurrence of severe disease and a high mortality, the CDC recommends respirators. The CDC policies are relatively stringent and may be influenced by the Occupations Health and Safety Administration (OSHA) recommendations. In the USA, the OSHA respiratory protection standard regulates the use of respirators at workplace. Under the regulation 29 CFR 1910.134, employers are required to provide respirators to the employees for protection from respiratory hazards [43]. The OSHA recommends using N95 or higher respirators for HCWs exposed to pandemic influenza [2] and SARS [44].

As highlighted in the results, the use of mask and respirator is not discussed in pandemic plans of some countries. Our findings corroborate with the WHO which identified during a comparative review of pandemic plans that only 33/76 (45%) of the national plans, discuss the role of masks, respirators and other PPEs [45]. Masks may be effective during early stages of a pandemic, when the mode of transmission and virulence characteristics are uncertain, and when pharmaceutical measure; such as a vaccine and/or antiviral, may not be available or delayed [2,46]. Studies have demonstrated that masks reduce shedding of virus from the wears month and could be as a mean of source control [47]. Therefore, mask use will not only protect HCWs but also prevent spread of infections from them to patients and other people surrounding them.

Uncertainty around the primary mode of transmission of influenza may be another reason contributing to the variations between the recommendations made by each country. Currently the relative contribution and significance of the each transmission mode is not known [48-50]. Most of the information regarding the mode of transmission of influenza is based on old experiments, observational studies during the outbreaks or on other in-direct research, for example drug and vaccine trials [46]. Droplet and contact is thought to be the main modes of transmission for seasonal influenza [2,22,51-53]. Droplet transmission is via large particles (typically > 5 um) that do not suspend in the air, while airborne transmission occurs through the dissemination of small virus containing particles (typically < 5 um) or droplet nuclei in the air. However some researchers argue that the evidence regarding droplet and contact being the main modes of transmission is not adequate [54] and there is more proof available in favor of the transmission of influenza through the aerosol mode [55-64]. Given the ongoing debate about the transmission, it is perhaps not surprising that none of the guidelines justify the selection of masks to evidence around influenza transmission.

Droplet and contact are thought to be primary modes of transmission of SARS [65], yet the use of respirators is highly recommended by the CDC and most of the countries in both low and high risk situations. In comparison, the WHO currently recommends masks for low risk situations and respirators for high risk. Low levels of evidence may be contributing to this difference. Most of the SARS guidelines are based on retrospective, observational studies conducted during the 2003–04 SARS outbreak. During that period, the WHO recommended HCWs to use respirator [66]. However, WHO updated its policy in 2007 and stated, “The current evidence suggests that SARS transmission in health care settings occurs mainly by droplet and contact routes. Therefore a medical mask is adequate for routine care”. The CDC, however, maintains its position and continues to recommend a respirator [1]. In the CDC guideline, the rationale of the airborne precautions for SARS is discussed in detail. Respirators are recommended due to the potential for the airborne transmission, frequently performed aerosol generating procedures (AGPs) and high case fatality among the HCWs. Unlike the WHO, the CDC discussed studies which favor airborne transmission of SARS [67].

There was also a lack of evidence based guidelines in regards to the use of masks/respirators when treating TB patients. The WHO quoted 13 studies on masks and respirator use for TB patients and concluded that there is little evidence on the effectiveness of respirators [30]. However the guideline states that “The available evidence, although weak and indirect, generally favors respirator use for protecting the wearer from TB”. High prevalence of TB in low income countries and increase chances of exposure due to respiratory aerosol in the healthcare facility setting could be an explanation for this recommendation. However, only the recommendations from Canada, Australia and China are aligned with the WHO and the CDC. Most of the low income countries recommended the use of respirators only when undertaking high risk procedures on patients with TB. Interestingly, the selective use of respirators when treating this patient group was also recommended in the UK policy. The UK recommendations have not been amended since 1994, when the British Thoracic Society (BTS) issued guidelines on the control and prevention of tuberculosis in the UK [68].

Regardless of the mode of disease transmission, all guidelines recommended the use of respirators while performing high risk procedures on influenza, SARS or TB patients. Studies have demonstrated that respiratory aerosols are produced more during AGPs. For example, the risk of influenza and SARS have been shown to increase after tracheal intubation and non-invasive ventilation [69,70] and risk of TB increases after bronchoscopy and sputum induction [71]. Therefore respirators are preferred during high risk procedures, as they filter small particles and designed to provide respiratory protection. Breathing air passes through the respirator filter and small respiratory aerosols are captured through diffusion and electrostatic mechanisms [72,73].

Training and fit testing are important components of a respiratory protection program and the efficacy of respirator use improves after being fit tested [74,75]. The risk of inhalation of infective particles is reduced if respirators are properly fitted to the face [64]. Although the WHO and the CDC discusses the role of fit testing in most of their guidelines, very few countries explain the procedure in detail. Guidelines from the low and middle income countries largely ignored this issue. Many of the guidelines reviewed also did not specify the maximum duration a single mask could be used for, while others varied in the times suggested. Advice pertaining to the reuse and extended of a mask/respirator was also not covered in most of the guidelines.

Even though the use/reuse of cloth masks is common, especially in low resource countries such as, for example in China [76] and Vietnam [77,78], none of the guidelines reviewed covered the use of these products. Currently, there is a lack of data to either support or refute the effectiveness of woven cloth masks in blocking influenza or virus transmission and fluid resistance. Regulatory standards require that surgical masks not permit blood or other potentially infectious fluids to pass through to or reach the wearer’s skin, mouth or other mucous membranes under normal conditions and for the duration of time that the protective equipment will be used. As it is not clear that cloth masks or improvised masks can meet the standards set by regulatory bodies and without better testing and more research, cloth masks or improvised masks generally have not been recommended as effective respiratory protective devices, or as devices to prevent exposure to splashes [72]. Currently there is no clinical trial data on the efficacy of cloth masks and most of the available studies are in-vitro [79-84]. Available evidence suggest that cloth masks may provide some protection, it is assumed to be considerable less when compared to the use of surgical masks and respirators [85]. However, it is theorized that some types of cloth fabric may provide better protection [86]. In a report by the National Institute of Health’s (NIH) Committee on the development of reusable facemasks for use during an influenza pandemic, the members were hesitant to discourage the use of cloth masks, but suggested caution around their use as they were not likely to be as protective as surgical masks or respirators [72].

This review has some limitations. Firstly, the guidelines from some countries could not be located, while others did not specifically address the use of masks and respirators. Secondly, while we tried to search for the most updated version of guidelines; some countries may have updated the documents and not made them publicly available. Finally, this study focused on selected high, middle and low income countries, but did not analyze every country. The situation may be different in these countries. For example, France recommends FFP2 and Austria recommends FFP3 for the HCWs in low and high risk situations during pandemics [22]. These policies are in line with the CDC policy. On the other hand, policies of the European CDC around the use masks and respirators are the same as those of the WHO [87].

## Conclusion

Health care organizations and countries have different policies and guidelines around mask and respirator use for influenza, SARS and TB. These policies not only vary regarding the choice of product used but also the application and specifications. These differences may reflect the relative lack of level-one evidence available to inform policy development. For the end user in a healthcare facility setting, the availability of conflicting guidance about mask use from different sources (such as WHO and in-country guidelines) may be confusing. Health organizations and countries should jointly evaluate the available evidence and develop a uniform policy on masks and respirator use in the health care setting. The situation in low income settings should be considered and various options should be explored. There is a need to conduct further studies to generate better evidence to inform policy and current practices. Currently there are major gaps around the modes of transmission of respiratory viruses, the efficacy of cloth masks and the impact of extended and re-use of masks/respirators.

## Competing interests

Professor Raina MacIntyre receives funding from influenza vaccine manufacturers GSK and CSL Biotherapies for investigator-driven research. Dr Holly Seale holds an NHMRC Australian based Public Health Training Fellowship (1012631). Payment for presentations: Dr Seale has received funding from Sanofi Pasteur, GSK and CSL Biotherapies for investigator driven research and for conference presentations.

## Authors’ contribution

AAC, HS and CRM contributed to the design of the study. AAC undertook the search strategy and made the initial selections which were subsequently validated by HS and CRM. AAC developed the first draft of the manuscript and HS and CRM extensively reviewed the paper. All authors read and approved the final manuscript.
